# Synthesis of porous high-temperature superconductors *via* a melamine formaldehyde sacrificial template †

**DOI:** 10.1039/d2na00333c

**Published:** 2022-06-16

**Authors:** Emily J. Luke, Jason Potticary, Lui R. Terry, Huan V. Doan, Roemer Hinoplen, Sam Cross, Valeska P. Ting, Sven Friedemann, Simon R. Hall

**Affiliations:** School of Chemistry, University of Bristol Cantock's Close Bristol BS8 1TS UK simon.hall@bristol.ac.uk; Bristol Composites Institute, Department of Mechanical Engineering, University of Bristol Queen's Building Bristol BS8 1TR UK; School of Physics, HH Wills Physics Laboratory Tyndall Avenue Bristol BS8 1TL UK

## Abstract

Nanostructured high-temperature superconductors YBa_2_Cu_3_O_6+*δ*_ and Bi_2_Sr_2_CaCu_2_O_8+*δ*_ were synthesised using a melamine formaldehyde sponge as a sacrificial template, *via* three solution-based approaches. In the case of YBa_2_Cu_3_O_6+*δ*_, a modified Pechini method produced a material with a superconducting transition at 92 K and a specific surface area of 4.22 m^2^ g^−1^. Further analysis with Hg porosimetry determined that the sponge exhibited a porosity of 82%. In the case of Bi_2_Sr_2_CaCu_2_O_8+*δ*_, this method produced a material that exhibited superconductivity at 86 K with a specific surface area of 9.62 m^2^ g^−1^. Hg-porosimetry determined that the BSCCO sponge exhibited a porosity of 78%.

## Abstract 2

The synthesis of porous high-temperature superconductors has been achieved for the first time using melamine formaldehyde as a sacrificial template.

## Graphical abstract

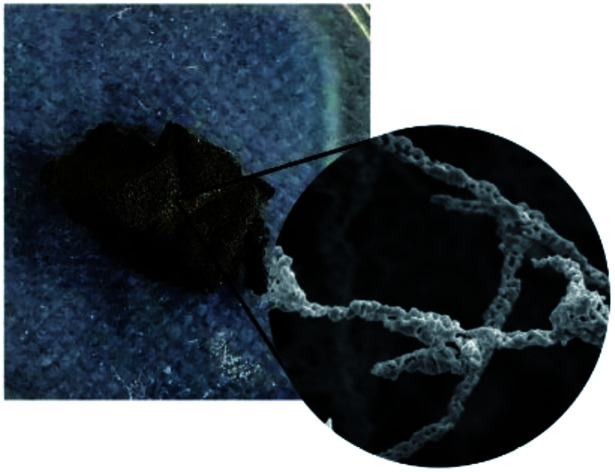

## Introduction

For materials with structural features at the nanoscale, certain physical phenomena such as catalytic capability, optical properties, and thermal conductivity can be altered and often enhanced.^1,2^ In the case of superconductors, mediating their nanostructures by the addition of nanorods has been shown to increase flux pinning and enhance the critical current density.^3^ The synthesis of a nanostructured and porous superconductor could allow it to be cooled more efficiently, as this would allow complete and rapid penetration of the material with the cryogen, preventing the formation of hot-spots within the sample at grain boundaries.^4,5^ Additionally, many superconductors require additional oxygenation steps during synthesis to ensure optimal doping, which can result in cracking and reduce mechanical stability.^6^ An intrinsically porous superconductor could potentially be able to be synthesised without the need for these additional oxygenation steps, as the diffusion pathway of oxygen through the sample would be much shorter due to the high surface area.^7^

Cuprate superconductors, such as YBa_2_Cu_3_O_6+*δ*_ (YBCO) and Bi_2_Sr_2_CaCu_2_O_8+*δ*_ (BSCCO), are commonly synthesised through traditional solid-state synthesis methods. BSCCO is part of a larger family of cuprate superconductors, which can result in various stoichiometric ratios of the form Bi_2_Sr_2_Ca_*n*−1_Cu_*n*_O_2*n*+4_ for *n* = 1, 2, 3.^8^ While solid-state syntheses are robust synthesis methods, the phase-purity of the product material is often limited by the need for long reaction times and poor control over the resultant particle morphology.^9^ This poor product quality is usually due to the lack of homogeneity in the starting material, and thus the reaction time of the synthesis is dictated by the rate of mass transport within the solid starting materials. For YBCO, this synthesis method can have adverse effects on the critical current density, generally due to grain boundaries impeding the flow of charge.^10,11^ For better control of macro- and nanomorphology, sol–gel techniques can be employed that utilise the homogeneous nature of solutions for the synthesis of solid-state materials. An example of this is the Pechini method, a technique that combines metal salts in the desired stoichiometric ratios, with a solution of ethylene glycol and citric acid.^12^ The ethylene glycol and citric acid can undergo an esterification reaction, forming a covalent polymer gel. This polymeric gel effectively chelates metal ions from solution, thereby enabling their homogeneous dispersion. Following this gelation phase, the material is calcined in a furnace, often at very high temperatures – for example YBCO can be calcined at 900 °C in the case of the Pechini synthesis.^12^ During this stage, the polymeric gel will undergo thermal degradation, leaving behind the inorganic material.

Additional solution-state methods of inorganic material synthesis include biomimetic syntheses, which have been applied to the synthesis of cuprate superconductors with great success.^13–15^ These methods utilise a natural biopolymer, such as dextran or sodium alginate, to chelate stoichiometric amounts of metal salts in solution. In these cases, the oxygen atoms in the biopolymer can chelate to the metal ions, forming a so-called egg-box conformation, which enables the biopolymer to behave similarly to the polymeric gel in the Pechini method.^16^ Intriguingly, biotemplated syntheses can be used to replicate the macrostructures of complex biological materials by using a biopolymer as a sacrificial template, enabling esoteric architectures such as superconducting pasta shapes and cuttlebones.^17,18^ YBCO sponges have been made previously through initial synthesis of non-superconducting Y_2_BaCuO_5_ foams and subsequent conversion to the superconducting YBCO phase.^19^

A relatively unexplored polymeric material for use as a template is melamine formaldehyde (MF). MF is a synthetic polymer formed by the polycondensation reaction between melamine and formaldehyde. This forms a resin that is used in commercial applications such as laminates, coatings and adhesives.^20^ MF can also be synthesised into a sponge morphology, for use as an abrasive material used for cleaning surfaces. There are many commercial MF sponges on the market, however the sponges used in this work are known as the ‘Flash Magic Eraser’ in the UK, and the ‘Mr Clean Magic Eraser’ in the USA. Within the scientific community, MF has been explored for its use as a flame retardant and as a device to separate oil from water.^21,22^ MF sponges are highly porous with 99% porosity and 100 μm pore sizes.^23^ More recently, MF sponges have been combined with sodium alginate and poly(imide dioxime), in order to extract uranium from seawater.^24^

In this work, for the first time we have synthesised superconducting sponges using MF as a template. We compare three different methods of synthesising superconducting sponge-like structures using MF sponges as sacrificial templates when applied to the two different cuprate superconductors, YBCO and BSCCO.

## Experimental details

### Materials

Yttrium nitrate hexahydrate (99.8%), calcium nitrate tetrahydrate (≥99%), strontium nitrate (>98%), bismuth nitrate pentahydrate (98%), barium nitrate (≥99%) and ethylene glycol (≥99%) were purchased from Sigma Aldrich, citric acid (99%) was purchased from Acros Organics, the Flash Magic Eraser was sourced from the FLASH store, and copper nitrate hemi(pentahydrate) (98–102%) was purchased from Alfa Aesar. Sodium alginate (Protonal GP 5450, medium G) was sourced from FMC Biopolymer. All materials were used as purchased without further treatment.

### Melamine formaldehyde sponge-templated synthesis

An aqueous solution of metal nitrates in stoichiometries matching the target phase was made – for YBCO this consisted of adding 0.1915 g yttrium nitrate hexahydrate, 0.2613 g barium nitrate, and 0.3489 g copper nitrate hemi(pentahydrate) to 10 mL deionised water and stirring overnight until all solids dissolved. For BSCCO, bismuth nitrate pentahydrate (0.4851 g), strontium nitrate (0.2116 g), calcium nitrate tetrahydrate (0.2361 g) and copper nitrate hemi(pentahydrate) (0.2326 g) were added to 10 mL water. To aid the dissolution of the bismuth nitrate pentahydrate, an extra 0.2 g ethylenediaminetetraacetic acid was added to the solution, which was then stirred at 80 °C until all powders had dissolved.

A section of the MF sponge approximately (2 × 2 cm) was submerged in *ca.* 5 mL of the aqueous precursor solution and degassed in a desiccator for 5 minutes. The sponge was then removed from the solution and dried at 80 °C overnight. The sample was then calcined at 900 °C with a 2 hour dwell time and a 5 °C min^−1^ ramp rate for YBCO and a 2 hour dwell time, 830 °C dwell temperature for BSCCO.

### Alginate-chelated synthesis

0.1 g sodium alginate was added to 50 mL water and stirred overnight until dissolved. MF sponge (approximately 2 × 2 cm) was submerged in this sodium alginate solution, and the sample was degassed under vacuum in a desiccator at room temperature for 5 minutes. The sponge was subsequently dried in an oven at 80 °C.

The alginate-penetrated sponge was added to an aqueous metal nitrate solution (as synthesised in the MF-only synthesis) degassed under vacuum in a desiccator for a further 5 minutes. After drying at 80 °C, the sponge was placed in a crucible and calcined with the same parameters as in the MF sponge-templated synthesis.

### Pechini method sponge synthesis

0.4306 g of citric acid was added to 10 mL ethylene glycol, so that the molar ratio was 1 : 80 citric acid : ethylene glycol, and the solution heated to 90 °C whilst stirring, until the citric acid dissolved. For YBCO: yttrium nitrate hexahydrate (0.1915 g), barium nitrate (0.2613 g), copper nitrate hemi(pentahydrate) (0.3489 g) were added, and then the solution stirred at 90 °C until the nitrates had fully dissolved. For BSCCO: bismuth nitrate pentahydrate, strontium nitrate, calcium nitrate tetrahydrate and copper nitrate hemi(pentahydrate) were added in a stoichiometric ratio of 2 : 2 : 1 : 2, bismuth : strontium : calcium : copper.

A section of MF sponge (approximately 2 × 2 cm) was submerged in this solution and placed under vacuum for 5 minutes to ensure full penetration of the sponge. The sponge was then warmed to 140 °C with a 2 hour dwell time to allow the ethylene glycol to polymerise with the citric acid and form a gel. Next, the sponge was placed in a crucible and calcined with the same parameters as in the MF sponge-templated synthesis.

### Characterisation

Powder X-ray diffraction (PXRD) was carried out on a Bruker D8 Advance with Cu-Kα, (*λ* = 1.5418 Å) source and a position-sensitive LynxEye Detector. Rietveld refinement was carried out with the open-source software Profex, which is a graphical interface for the refinement software, BGMN.^25^ Scanning electron microscopy (SEM) was carried out on a JEOL IT300. For YBCO resistance measurements, the sample was etched with argon plasma for 15 seconds, before gold contacts were evaporated on top of the sample. Gold wires were contacted to the gold contacts with silver paint (Dupont N4929), before a 4-probe AC method was used to measure the resistance with an applied current of 10 μA. For BSCCO, the gold wires were attached directly to the sponge with silver paint (Dupont N4929) before the 4-probe AC method was used to measure the resistance. Gas sorption isotherms were determined using nitrogen sorption at 77 K with a Micromeritics 3-Flex volumetric gas sorption analysis system (nitrogen with purity of 99.9999% was purchased from Air Products). 100 mg of samples were degassed under dynamic high vacuum (10^−6^ mbar) at 120 °C over 6 hours prior to analysis. Helium was used for free space determination following isothermal data collection. Hg-porosimetry was carried out by MCA Services on a MicroActive AutoPore V 9600.

## Results and discussion

**Fig. 1:**
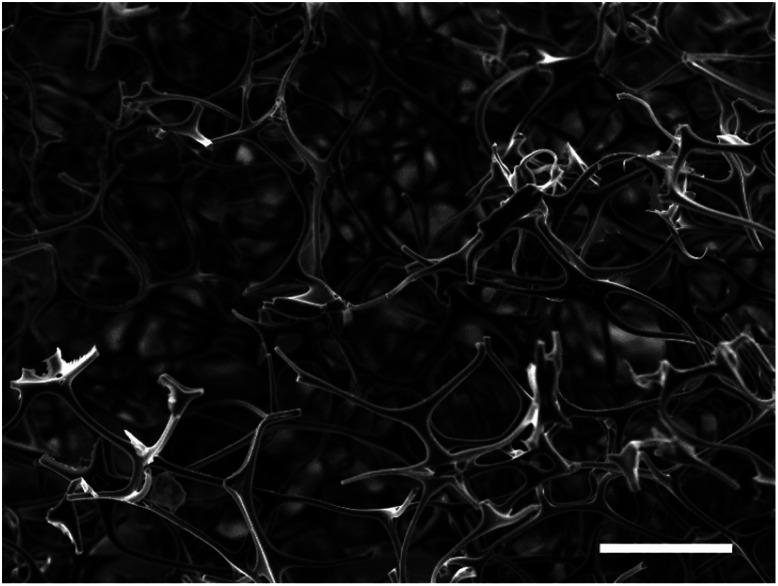
Scanning electron micrograph of the original MF sponge. The sample was sputtered with a 15 nm coating of silver but was otherwise untreated. Scale bar indicates a length of 100 μm.

Scanning electron microscopy (SEM) on the MF sponge was used to determine its initial structure. From Fig. 1, it can be seen that the sponge was highly porous and exhibited an open nanostructure.

**Fig. 2:**
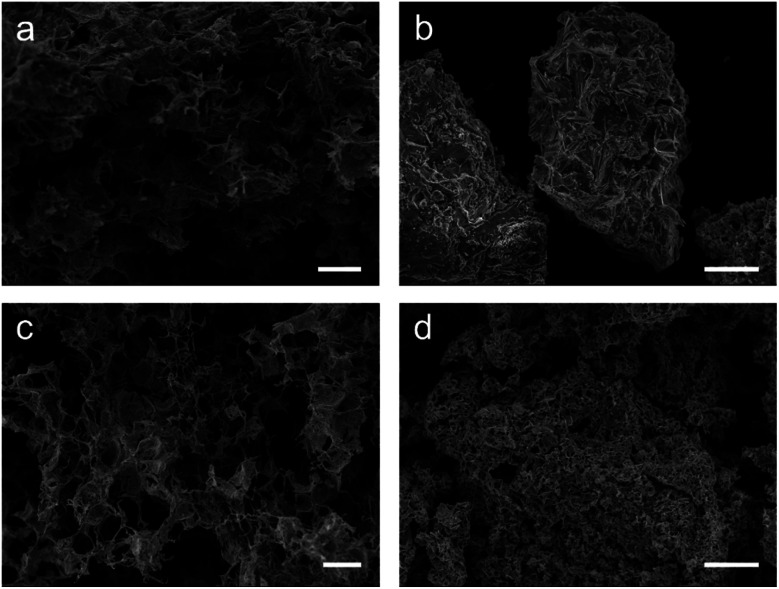
Scanning electron micrographs of the calcined sponges (a) the YBCO sample with only the MF sponge. (b) The BSCCO sample with only the MF sponge. (c) The YBCO with the sodium alginate biopolymer. (d) The BSCCO with the sodium alginate biopolymer. Scale bars indicate a length of (a), (c) 100 μm, and (b), (d) 50 μm.

The first method attempted was carried out by simply soaking the MF sponge in an aqueous precursor solution containing the metal salts in the correct stoichiometric ratios for the target phase and subsequent calcination (samples labelled hereafter as MF). The resulting MF-YBCO sample did exhibit a sponge-like morphology, shown in Fig. 2a, appearing to resemble the original MF structure. However, the MF-BSCCO sample did not exhibit a sponge-like morphology, shown in Fig. 2b. Instead, it appeared to form large amorphous clusters of non-porous material.

**Fig. 3:**
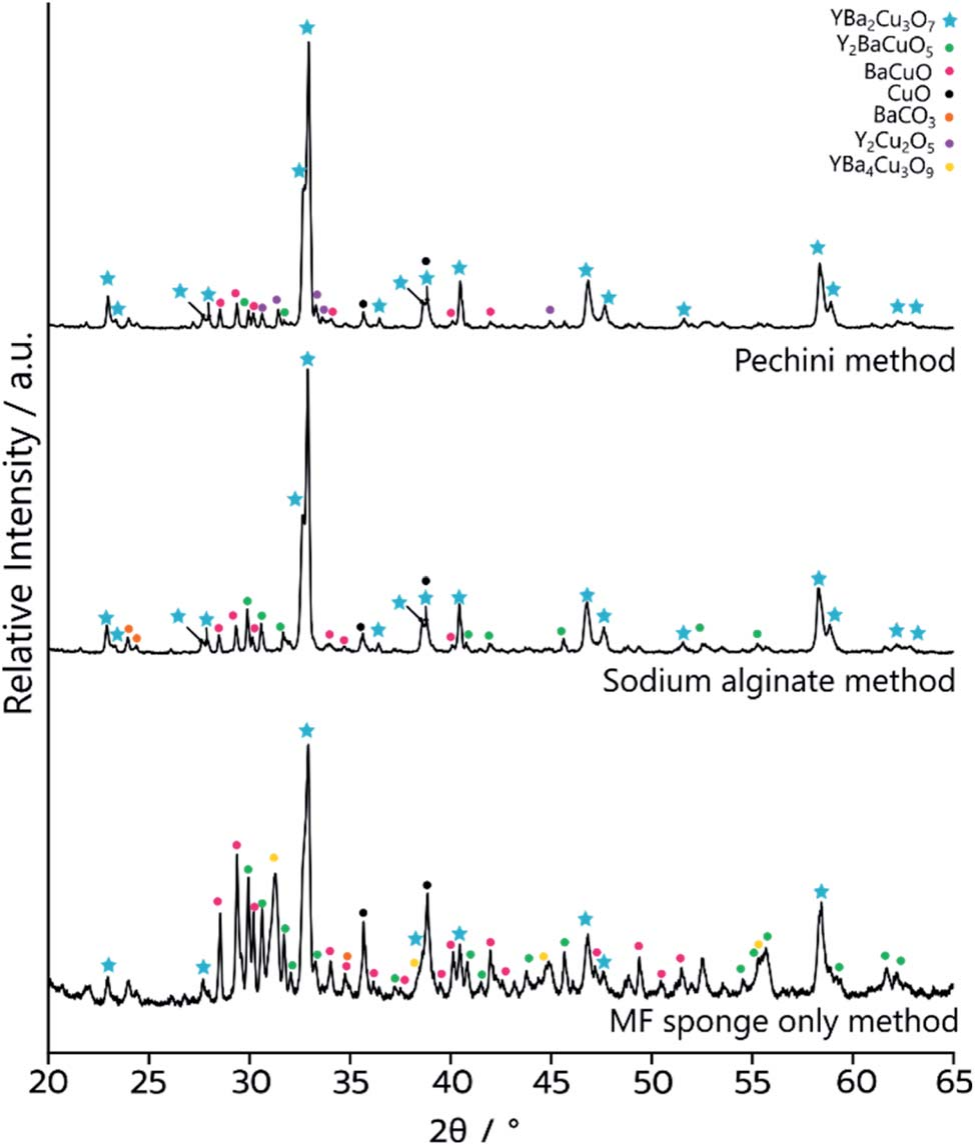
PXRD data of the YBCO sponge samples after calcination. Bottom – the sample synthesised by templating to MF sponge only, MF-YBCO. Middle – the sample synthesised by templating to sodium alginate and the MF sponge, MFA-YBCO. Top – the sample synthesised through the Pechini method and MF sponge, MFP-YBCO.

The as synthesised MF-YBCO sample also exhibited a slight green colour in some places, visibly indicating the presence of a common impurity of YBCO, Y_2_BaCuO_5_ (Y211). Y211 is a so-called ‘green phase’ which is not superconducting.^26^ The presence of the Y211 phase and target YBCO phase, YBa_2_Cu_3_O_7−*δ*_, were confirmed by PXRD, shown in Fig. 3. Rietveld refinement determined that the sample consisted of only 36% of the target YBCO phase, YBa_2_Cu_3_O_7−*δ*_. A summary of the constituent phases within the sample as determined by Rietveld refinement can be found in Table 1.

**Table 1:** Approximate phase quantities of the different products of the three different synthesis methods for YBCO, extracted *via* refinement of the PXRD patterns

Phase	Quantity		
	MF only/%	Alginate method/%	Pechini method/%
YBa_2_Cu_3_O_6+*δ*_	36	65	69
Y_2_BaCuO_5_	17	13	4
Ba_0.98_CuO_2.07_	18	8	9
Y_2_Cu_2_O_5_	4	1	8
CuO	12	9	7
BaCO_3_	4	4	3
YBa_4_Cu_3_O_9_	9	0	0

The MF-BSCCO sample was relatively pure despite lacking the adoption of the nanostructure, producing 86% of the target phase (Fig. 2c and Table 2).^27^ It could therefore be inferred that the MF sponge behaves as a good chelating agent to the metal ions in solution, thus promoting a purer product. It appears then, MF could be a potential synthetic avenue for the sol–gel synthesis of BSCCO. The addition of EDTA as a chelating agent to aid in dissolution of Bi(NO_3_)·5H_2_O may also have assisted with improving the purity of the material. The crystal structure used for refinement in all three BSCCO samples examined in this paper was for Bi_2.09_Sr_0.9_CaCu_2_O_8+*x*_ as it provided the best refinement fit. This is a modulated structure of conventional BSCCO.^27^

**Table 2:** Approximate phase quantities of the products of the three different synthesis methods for BSCCO, extracted using refinement of the PXRD patterns

Phase	Quantity		
	MF only/%	Alginate method/%	Pechini method/%
Bi_2.09_Sr_0.9_CaCu_2_O_8+*x*_	86	65	56
Bi_10_Sr_10_Cu_5_O_29_	6	17	16
Sr_6.27_Ca_4.73_Bi_6_O_22_	6	5	14
CaCO_3_	2	14	11
CuO	0	0	3

Next, syntheses for both YBCO and BSCCO were carried out using the biopolymer sodium alginate in addition to the MF sponge scaffold (calcined samples herein labelled with MFA). MFA-YBCO had an increased purity over MF-YBCO, with 65% consisting of the target phase. However, for these samples the resultant MFA-YBCO sponge was extremely fragile and the MFA-BSCCO sample was porous although it did not result in a sponge-like morphology. This can be seen in Fig. 2c and d, for MFA-YBCO and MFA-BSCCO respectively. The reason for the fragility of MFA-YBCO can be inferred from the scanning electron micrograph, as the pore walls of the sample are thin. The fragility could be due to the sodium alginate only forming a thin layer on top of the MF sponge framework. As such, the resultant superconductor is likely to form a hollow shell around the MF sponge, thus leading to an extremely friable material.

**Fig. 4:**
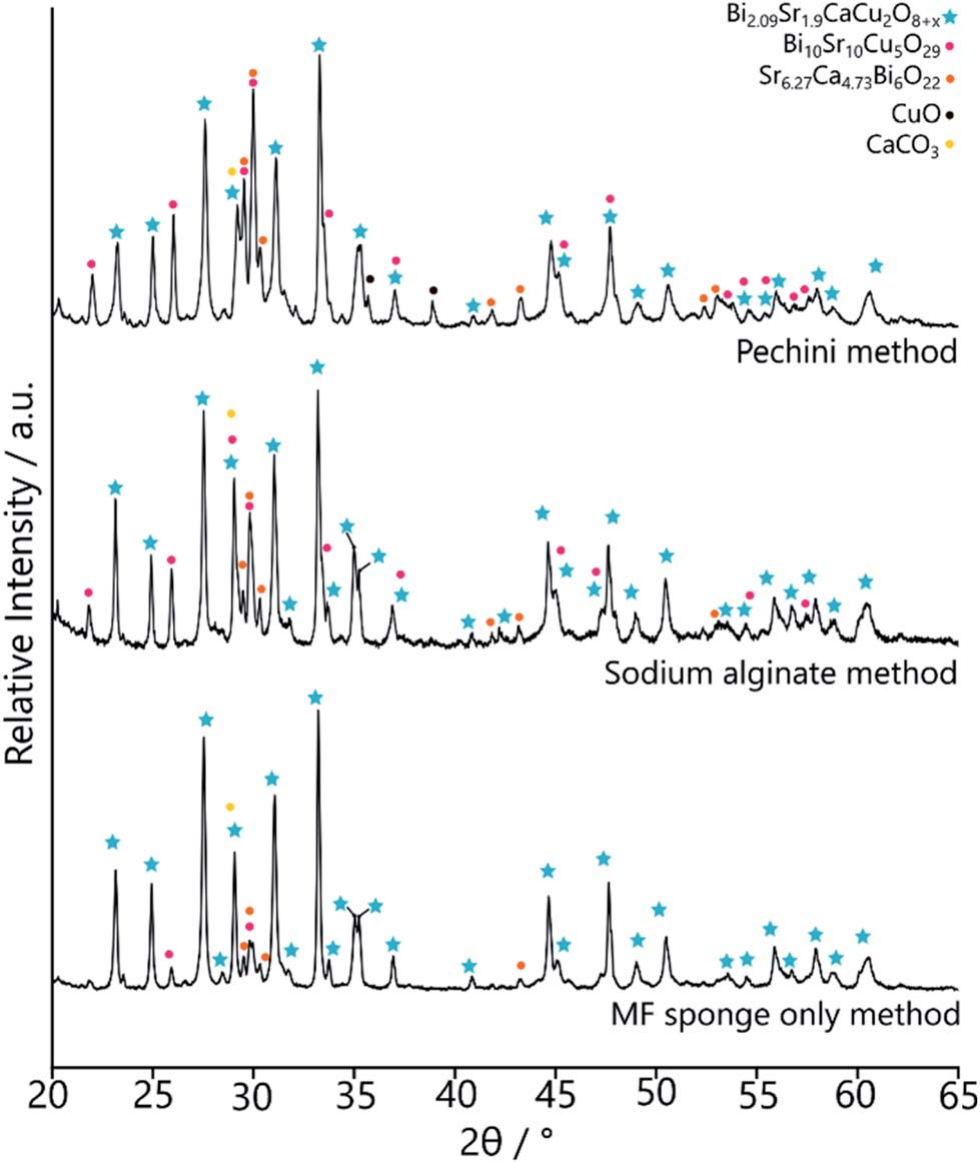
PXRD data of the BSCCO samples after calcination. Bottom – the sample synthesised by templating to MF sponge only, MF-BSCCO. Middle – the sample synthesised by templating to sodium alginate and the MF sponge, MFA-BSCCO. Top – the sample synthesised through the Pechini method and MF sponge, MFP-BSCCO.

The MFA-BSCCO sample was less pure with the addition of sodium alginate when compared to the MF synthesis first attempted, as shown in Fig. 4. Rietveld refinement determined that the material synthesised with the alginate contained 65% of the target phase. MFA-BSCCO showed no evidence of an extensive sponge-like morphology, though there was evidence of small pores on the surface of the material. To determine if the addition of sodium alginate was the cause of the lower purity of the sample, a control sample with no MF sponge present was synthesised. In this case, Na_3_Ca_2_BiO_6_ was one of the primary phases present (see ESI, Fig. S9 and Table S2†) and the desired BSCCO phase was not detectable.

**Fig. 5:**
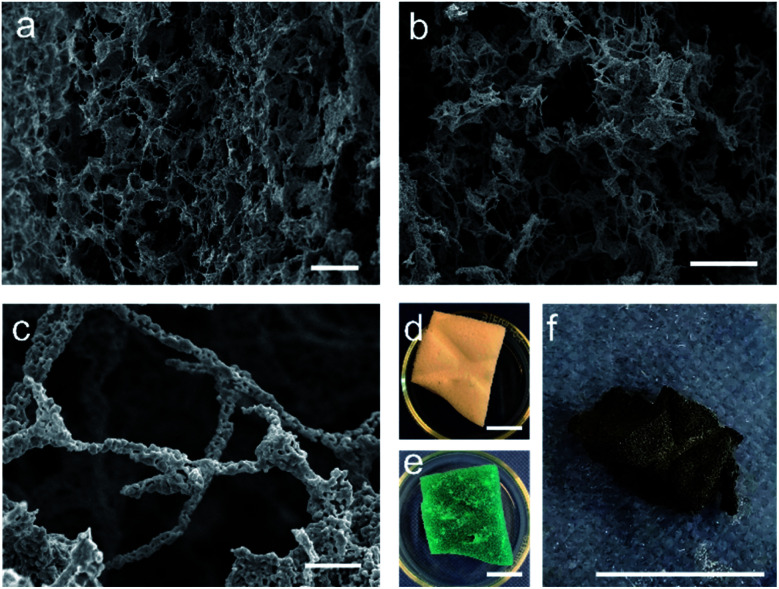
(a)–(c) SEM micrographs of the MFP-YBCO sponge-like material. It can be seen from (a) and (b) that the sample is formed of an open sponge-like network. (c) Shows that the MFP-YBCO struts are formed by reticulated crystallites. (d) Image of the sponge with the additional decorative branding (e) the sponge after being soaked in YBCO Pechini solution and gelled. (f) The MFP-YBCO sponge after being calcined in the furnace, the decorative branding of the sponge is still visible, showing that the synthesis can replicate the macro-structure of the MF sponge. Scale bars in (a) and (b) are 100 μm, (c) is 10 μm. (d)–(f) are 1 cm.

As the two attempted synthesis methods did not yield both a high purity of target phase and good retention of nanostructure, syntheses using a modified Pechini method were then attempted (herein labelled MFP). The Pechini method has previously been employed for the synthesis of both YBCO and BSCCO, but we combined it with the MF sponge in this case in an attempt to replicate the sponge nanostructure.^12,28^ It was thought that the polymeric gel formed would enable the replication of the sponge-like structure after the calcination of the sample had been carried out. In this case, the amount of citric acid added was much less than the conventional Pechini method, as the sponge often degraded upon the gelation step with the usual amount of citric acid. After calcination, the decorative branding of the sponge was still visible on the sample, as is shown in Fig. 5f. For MFP-YBCO, it can be seen that the sample exhibits a highly porous morphology, similar to that of the alginate sample, and exhibits small crystallites of YBCO in a reticulated structure (Fig. 5c) The connecting struts of YBCO are also thicker than those found in the MFA-YBCO sample, however, indicating that the sample could be more mechanically stable than MFA-YBCO. This was qualitatively confirmed, as the sample did not collapse upon sputter treatment during SEM preparation.

PXRD (Fig. 3) of MFP-YBCO indicated that, while the Y211 and BaCuO phases were still present, the major phase was the target YBCO phase. Rietveld analysis of the samples confirmed that the material was 69% of the target YBCO (Y123) phase. A summary of all the phase information for the MF Sponge YBCO experiments as refined by Rietveld refinement can be found in Table 1.

**Fig. 6:**
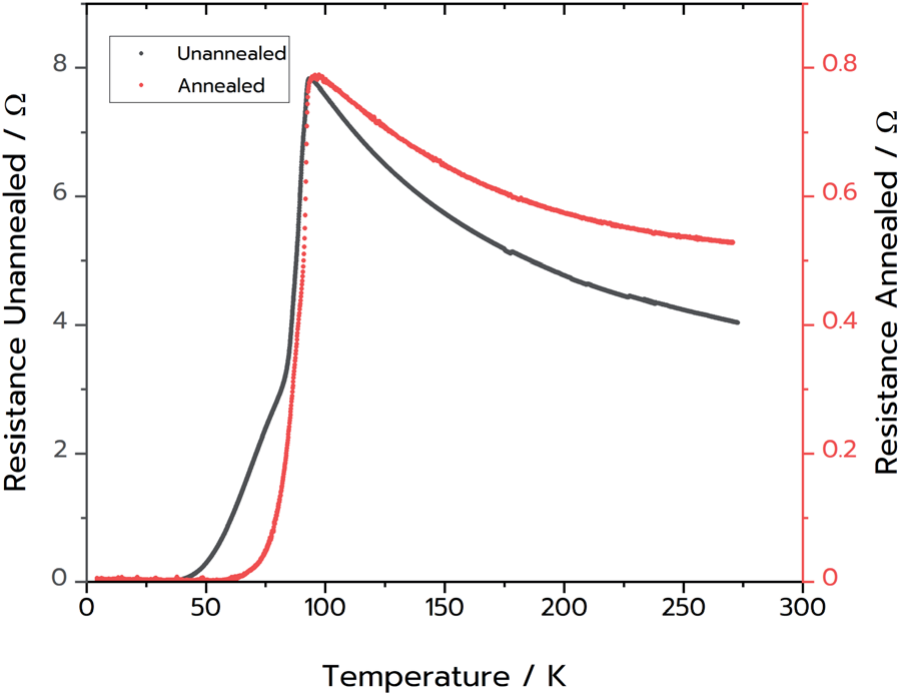
Black: two-point resistance measurements on the MFP-YBCO sample. Red: four-point resistance measurement on the MFP-YBCO sample after annealing in oxygen for 48 hours at 500 °C. Resistance data for both samples were measured from 4.5 K up to room temperature.

Resistance measurements confirmed that MFP-YBCO is able to superconduct (Fig. 6). The resistance against temperature graph shows that MFP-YBCO had an onset critical temperature (*T*_C_) of 92 K. This is comparable to the *T*_C_ typically exhibited by YBCO, which is 93 K.^29^ The transition was relatively broad compared to that of a single crystal, and exhibited a shoulder at 70 K. Annealing at 500 °C in an atmosphere of oxygen for 48 hours removed this shoulder and sharpened the transition.

**Fig. 7:**
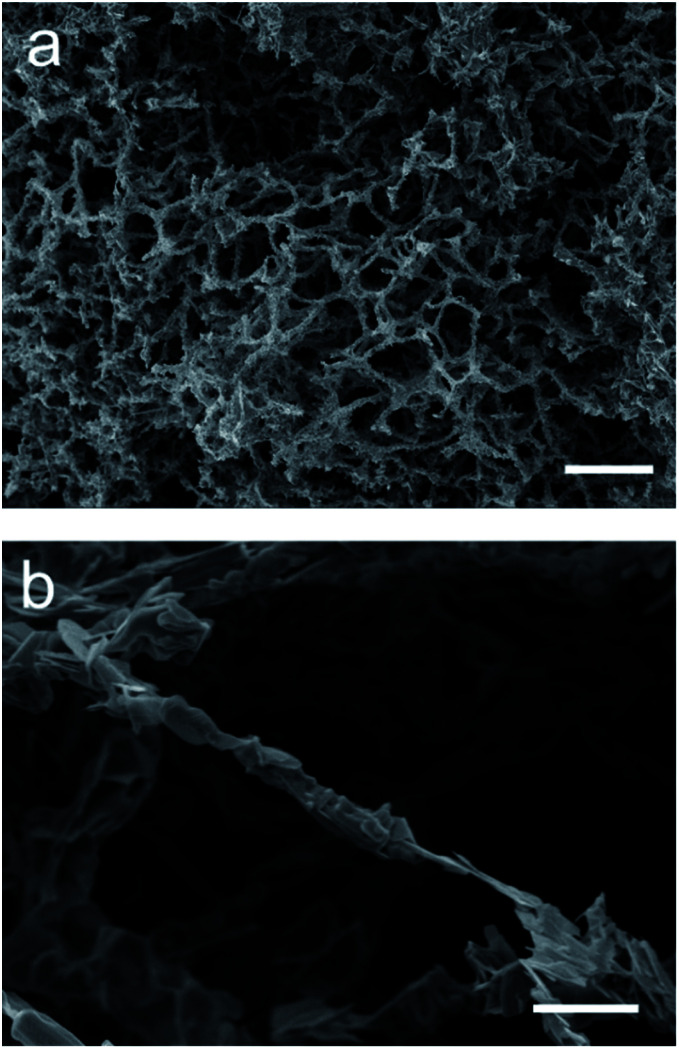
(a) and (b) SEM micrographs of the MFP-BSCCO sponge-like material. It can be seen from (a) that the sample is formed of an open sponge-like network. (b) Shows that the structure is still reticulated, however the crystallites in this case are more anisotropic than the crystallites in MFP-YBCO. Scale bars in (a) 100 μm (b) 10 μm.

**Fig. 8:**
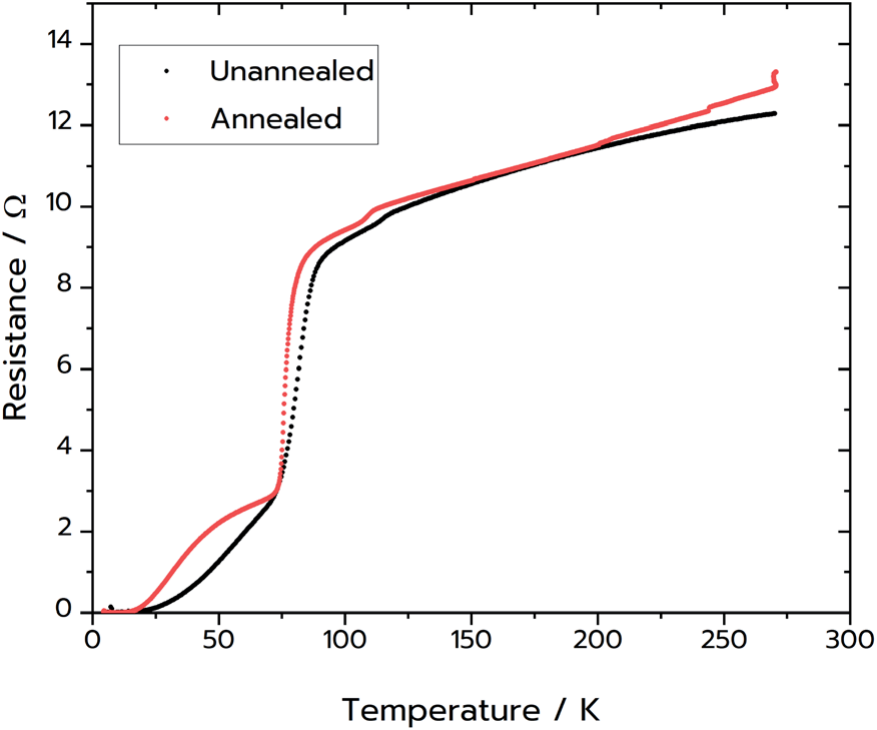
(Black) Four-point resistance measurements on the MFP-BSCCO sample. (Red) Four-point resistance measurements on sample of MFP-BSCCO that had been annealed at 830 °C for 24 hours. For this sample the gold wires were attached directly to the sponge with silver paint. Resistance for both samples was measured from 4.5 K up to room temperature.

Following the success of the Pechini synthesis with YBCO, the method was also attempted with BSCCO, hereafter labelled as MFP-BSCCO. This also resulted in an open sponge-like structure (Fig. 7), however the crystallites of the material were more plate-like than when compared to the MFP-YBCO sample. MFP-BSCCO has a larger and more anisotropic unit cell than MFP-YBCO, so it is not unreasonable that this would be the cause of the difference in crystallite morphology.^30^ PXRD (Fig. 4) shows that the MFP-BSCCO sample is less pure than the MFP-YBCO sample, with an overall fraction of 56% of the target phase. Four-point resistance measurements were carried out on the sponge (Fig. 8), and it was determined that the material had an onset *T*_C_ of 86 K. The BSCCO family has a large range of potential *T*_C_'s depending on the stoichiometry of the phase, with 20 K for Bi_2_Sr_2_CuO_6_, 85 K for Bi_2_Sr_2_CaCu_2_O_8_ and 110 K for Bi_2_Sr_2_Ca_2_Cu_3_O_10_.^31^ In this case, therefore, the onset *T*_C_ reported in this work of 86 K does align with what would be expected of the target phase, Bi_2_Sr_2_CaCu_2_O_8_. The sample was also annealed in air for 24 hours at 830 °C in an attempt to sharpen the superconducting transition.^32^ While the transition was sharper, the overall *T*_C_ decreased to 82 K, and the shoulder caused by impurities became more pronounced. Further refinement of the annealing parameters would need to be undertaken to fully understand why this is the case.

Finally, surface area and porosity measurements were carried out on both MFP samples to fully characterise the porous nature of the sponges and to ensure that the sponge morphology was expressed throughout the material. To evaluate the surface area and presence of micropores or mesopores, N_2_ gas sorption measurements (77 K) were conducted on the materials (Fig. S11a and b†). For both materials, very low uptakes of N_2_ were observed indicating the sponges have very limited micro and mesopore volume and surface area. A type IV isotherm with a hysteresis between the adsorption and the desorption branches was observed for MFP-YBCO sponges, indicating that a small proportion of mesopores (diameters 2–50 nm) were present in the sample. This hysteresis loop was classified as type H4 and closed at *P*/*P*_0_ ∼ 0.4, suggesting that there was not a significant volume of slit-like pores accessible to N_2_ below this width (∼2.2 nm) and that larger voids were wedge shaped. The pore size distribution for MFP-YBCO (Fig. S11c†) was calculated using the Barrett–Joyner–Halenda (BJH) method, displaying a broad distribution of mesopores in the sponges with a modal pore size of 4.8 ± 1.3 nm. However, with a cumulative pore volume of 0.01 cm^3^ g^−1^, the level of mesopores in the sample is very low and likely that the majority of the materials porosity comes from macropores (>50 nm) as observed *via* SEM. In contrast, MFP-BSCCO sponges exhibited a type III isotherm with a reversed curvature indicating that the interaction between this sample and nitrogen is relatively weak and likely that this sponge also consisted of mainly macropores, as observed by SEM. Due to the low interaction, an accurate pore size distribution could not be extracted for this sample.

Brunauer–Emmett–Teller (BET) surface area measurements (Fig. S11†) revealed that both samples exhibited low surface areas, ∼4.22 m^2^ g^−1^ and ∼9.62 m^2^ g^−1^ for MFP-YBCO and MFP-BSCCO samples respectively, in agreement with the low level of micro and mesopores. Despite the observation of a porous sample by SEM, both samples consisted of mostly large voids that are connected by wires that wouldn't contribute significantly to the surface area of the sample.

**Fig. 9:**
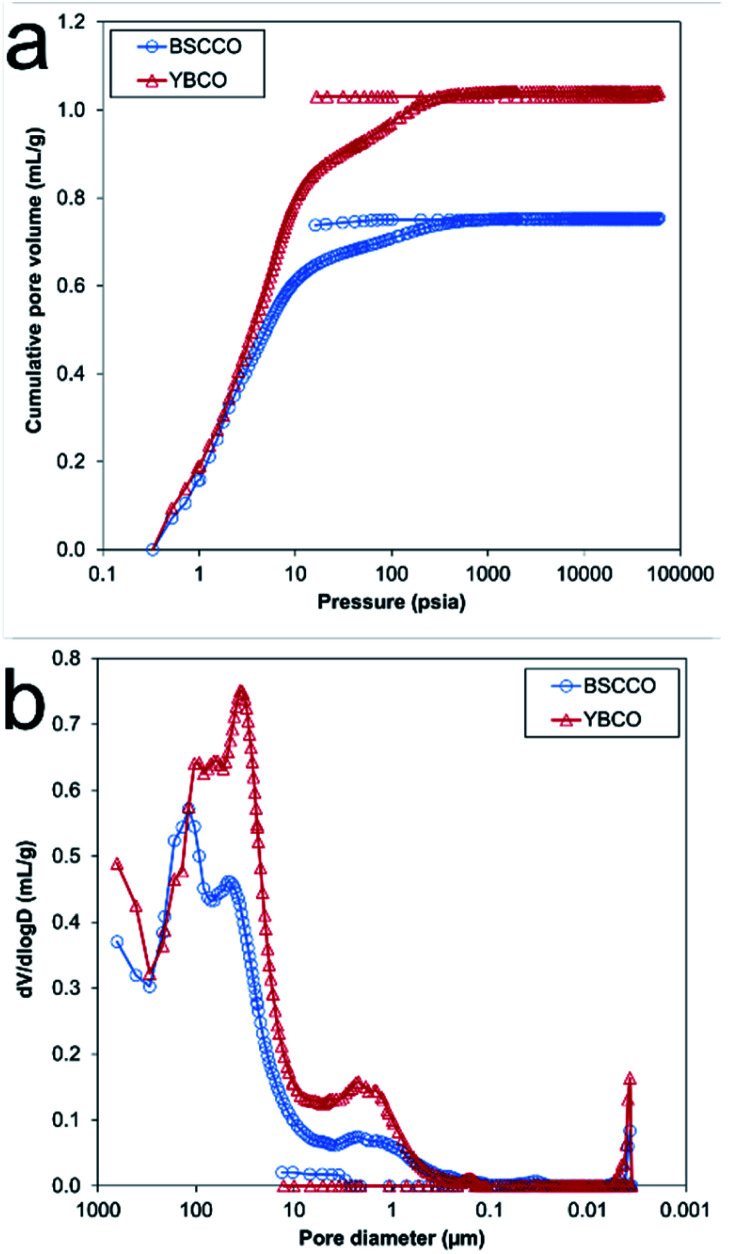
Overlay plots for the MFP-YBCO and MFP-BSCCO sponges. (A) Cumulative pore volume data from Hg-porosimetry experiments with BSCCO (blue) and YBCO (red). (B) Differential pore volume distribution against pore diameter of YBCO (red) and BSCCO (blue).

To assess the macroporous character of the samples, Hg-porosimetry was carried out. The observed cumulative pore volume and pore size distribution can be found in Fig. 9. Several different sizes of macropores (pores > 50 nm in diameter) were found throughout the samples. For MFP-YBCO, a macropores of 2.3 μm, 37 μm and 104 μm width were observed. For MFP-BSCCO, a similar macropore size range was observed of 2.2 μm, 43 μm and 118 μm. In agreement with the nitrogen isotherms, there were virtually no pores < 50 nm in the samples. MFP-YBCO exhibited a larger total pore volume than MFP-BSCCO, resulting in a higher porosity, 82% and 78% respectively.

These data indicate that the Pechini method exhibits the best compromise between sample purity and replication of sponge morphology of the three techniques. While the sodium alginate method allowed for the replication of the sponge morphology for YBCO, this was not the case for BSCCO, making the Pechini method the more ubiquitous method.

## Conclusions

In this paper, we compared three ways of using a MF sponge as a sacrificial template in the synthesis of two high-temperature superconductors: YBCO and BSCCO. Of these three methods the most robust was the Pechini method, as it resulted in full replication of the sponge morphology of the original MF template and reasonably high purity for both YBCO and BSCCO.

Despite poor replication of the nanostructure, the MF-BSCCO synthesis yielded the highest purity sample from the different methods attempted. This method could therefore be adapted in the future, as a new general method for the sol–gel synthesis of BSCCO, thus yielding high quality BSCCO samples despite short dwell times.

YBCO samples were successfully synthesised with a sponge-like morphology with a predominantly macroporous structure. Samples synthesised by the Pechini method were shown by direct resistance measurements to be superconductive with a critical temperature of 92 K and were relatively pure (69% of the target phase) as shown by the powder XRD analysis. These samples were far less fragile than the samples synthesised with sodium alginate, and showed a reticulated morphology when examined with SEM. Similarly, the Pechini method was compatible with the synthesis of BSCCO, resulting in a sponge that containing anisotropic crystallites and a phase purity of 55%. This sample was also superconducting, with a critical temperature of 86 K. Despite low overall surface area as determined by N_2_ gas adsorption measurements, these samples were both found to be macroporous samples, with high porosities by Hg-porosimetry.

These sponge structures are polycrystalline, so could be limited in terms of critical current density due to the nature of the cuprate superconductors. This work does however highlight the use of MF as a structure-directing agent for the synthesis of cuprate superconductors, suggesting a new generalised strategy for synthesising these materials with good control over the macrostructures and phase composition. Additionally, we have developed a novel way of synthesising porous superconductors, potentially enabling access to the rapid cooling of superconductors in the future. We envisage that this method would have broader applicability in the synthesis of other porous functional materials.

## Data availability

All underlying data are provided in full within in this paper.

## Author contributions

S. R. H. initiated and supervised the project. E. J. L., J. P. performed the synthesis and structural characterisation experiments at Bristol. L. R. T. and H. V. D. carried out the porosity characterisation in Bristol. R. H. and S. C. assisted with the annealing parameters for, and resistance measurements of the MFP samples. V. P. T. and S. F. provided additional supervision and discussion. All authors contributed to the discussion of the results, analysis of the materials and to manuscript preparation.

## Conflicts of interest

There are no conflicts to declare.

## Supplementary Material

NA-004-D2NA00333C-s001

## References

[cit1] Yina Y., Talapin D. (2013). Chem. Soc. Rev..

[cit2] Li Z., Wang L., Li Y., Feng Y., Feng W. (2019). Compos. Sci. Technol..

[cit3] Yang P., Lieber C. M. (1997). J. Mater. Res..

[cit4] Tournier R., Beaugnon E., Belmont O., Chaud X., Bourgault D., Isfort D., Porcar L., Tixador P. (2000). Supercond. Sci. Technol..

[cit5] Gokhfeld D. M., Koblischka M. R., Koblischka-Veneva A. (2020). Phys. Met. Metallogr..

[cit6] Diko P. (2004). Supercond. Sci. Technol..

[cit7] Koblischka M. R., Koblischka-Veneva A. (2018). AIMS Mater. Sci..

[cit8] Chu C. W., Deng L. Z., Lv B. (2015). Phys. C.

[cit9] Danks A. E., Hall S. R., Schnepp Z. (2016). Mater. Horiz..

[cit10] Pathak L. C., Mishra S. K. (2005). Supercond. Sci. Technol..

[cit11] Hilgenkamp H., Mannhart J. (2002). Rev. Mod. Phys..

[cit12] Motta M., Deimling C. V., Saeki M. J., Lisboa-Filho P. N. (2008). J. Sol-Gel Sci. Technol..

[cit13] Hall S. R. (2006). Adv. Mater..

[cit14] Walsh D., Wimbush S. C., Hall S. R. (2009). Supercond. Sci. Technol..

[cit15] Green D. C., Boston R., Glatzel S., Lees M. R., Wimbush S. C., Potticary J., Ogasawara W., Hall S. R. (2015). Adv. Funct. Mater..

[cit16] Morris E. R. (1986). Br. Polym. J..

[cit17] Green D. C., Lees M. R., Hall S. R. (2013). Chem. Commun..

[cit18] Culverwell E., Wimbush S. C., Hall S. R. (2008). Chem. Commun..

[cit19] Reddy E. S., Herweg M., Schmitz G. J. (2003). Supercond. Sci. Technol..

[cit20] Merline D. J., Vukusic S., Abdala A. A. (2013). Polym. J..

[cit21] Ruan C., Ai K., Li X., Lu L. (2014). Angew. Chem., Int. Ed..

[cit22] Chen X., Weibel J. A., Garimella S. V. (2016). Ind. Eng. Chem. Res..

[cit23] Feng Y., Yao J. (2018). Ind. Eng. Chem. Res..

[cit24] Wang D., Song J., Lin S., Wen J., Ma C., Yuan Y., Lei M., Wang X., Wang N., Wu H. (2019). Adv. Funct. Mater..

[cit25] Doebelin N., Kleeberg R. (2015). J. Appl. Crystallogr..

[cit26] Kanoda K., Takahashi T., Kawagoe T., Mizoguchi T., Kagoshima S., Hasumi M. (1987). Jpn. J. Appl. Phys..

[cit27] Gladyshevskii R. E., Flükiger R. (1996). Acta Crystallogr. Sect. B Struct. Sci..

[cit28] Peng Z. S., Hua Z. Q., Li Y. N., Di J., Ma J., Chu Y. M., Zhen W. N., Yang Y. L., Wang H. J., Zhao Z. X. (1998). J. Supercond. Nov. Magnetism.

[cit29] Liang R., Dosanjh P., Bonn D. A., Baar D. J., Carolan J. F., Hardy W. N. (1992). Phys. C Supercond..

[cit30] MacManus-Driscoll J. L., Wimbush S. C. (2011). IEEE Trans. Appl. Supercond..

[cit31] Özçelik B., Gürsul M., Sotelo A., Madre M. A. (2015). J. Mater. Sci. Mater. Electron..

[cit32] Khalil S. M., Sedky A. (2005). Phys. B Condens. Matter.

