# Supplementary material for: Synthesis of porous high-temperature superconductors via a melamine formaldehyde sacrificial template
Source: Nanoscale Adv. 2022 Jun 16;4(14):3101–8. doi: 10.1039/d2na00333c (PMC9418839; doi:10.1039/d2na00333c)
Supplement: NA-004-D2NA00333C-s001 [file NA-004-D2NA00333C-s001.pdf]

Electronic Supplementary Material (ESI) for Nanoscale Advances.
This journal is © The Royal Society of Chemistry 2022

*Supplementary information for*

# Synthesis of porous high-temperature superconductors *via* a melamine formaldehyde sacrificial template

Emily J. Luke[1], Jason Potticary[1], Lui R. Terry[2], Huan V. Doan[1], Roemer Hinoplen[3], Sam Cross[3], Valeska P. Ting[2], Sven Friedemann[3], Simon R. Hall[1*]

Affiliations:

[1] School of Chemistry, University of Bristol, Cantock's Close, Bristol, BS8 1TS

[2] Bristol Composites Institute, Department of Mechanical Engineering, University of Bristol, Queen's Building, Bristol, BS8 1TR

[3] School of Physics, HH Wills Physics Laboratory, Tyndall Avenue, Bristol, BS8 1TL

*email: simon.hall@bristol.ac.uk

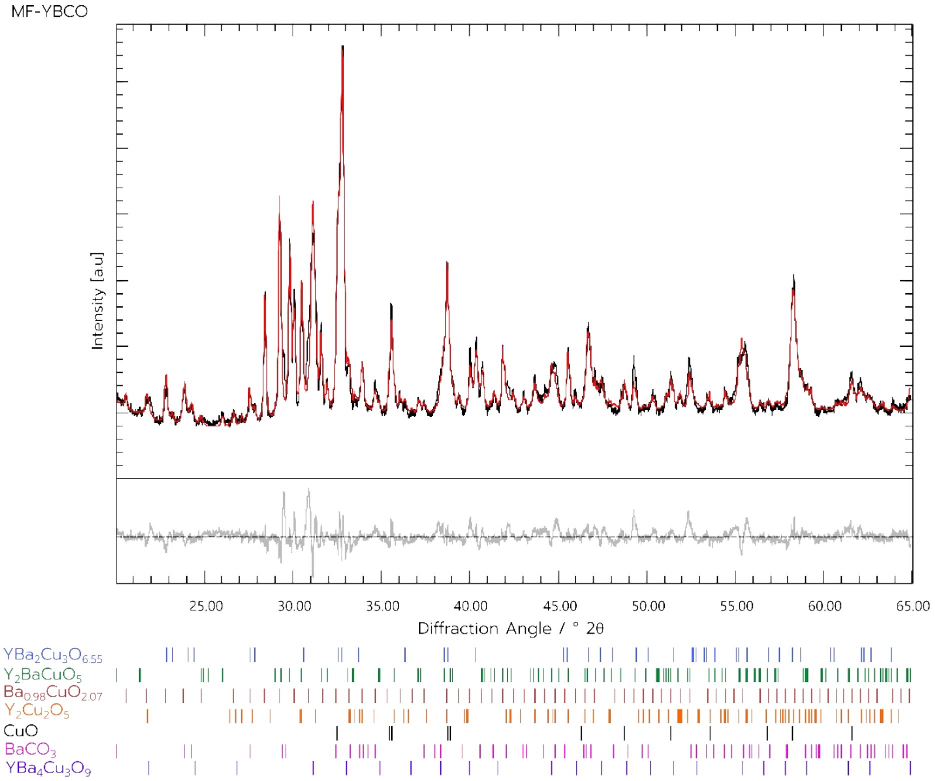


**Figure S1** – YBCO MF sponge only (MF-YBCO), refined PXRD pattern with $R_{wp}$ = 6.93 %. Observed data points and calculated pattern are shown as black and red, respectively.

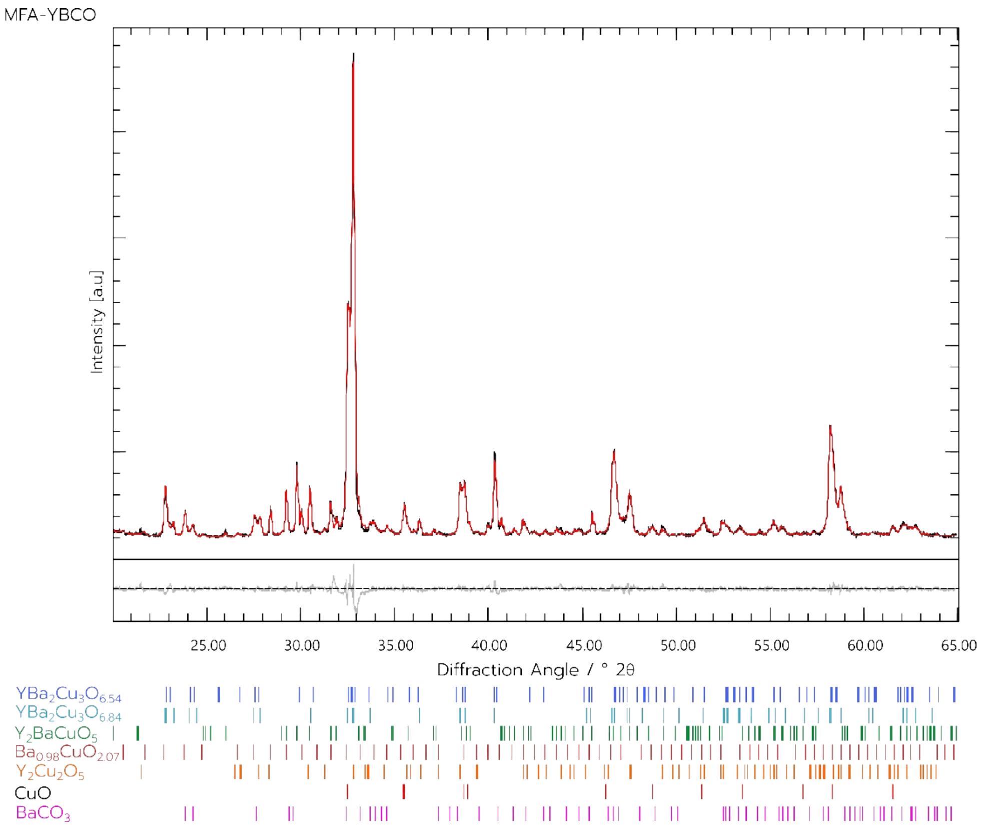


**Figure S2** – YBCO sodium alginate only (MFA-YBCO), refined PXRD pattern with $R_{wp}$ = 4.01 %. Observed and calculated plots are shown as black and red, respectively.

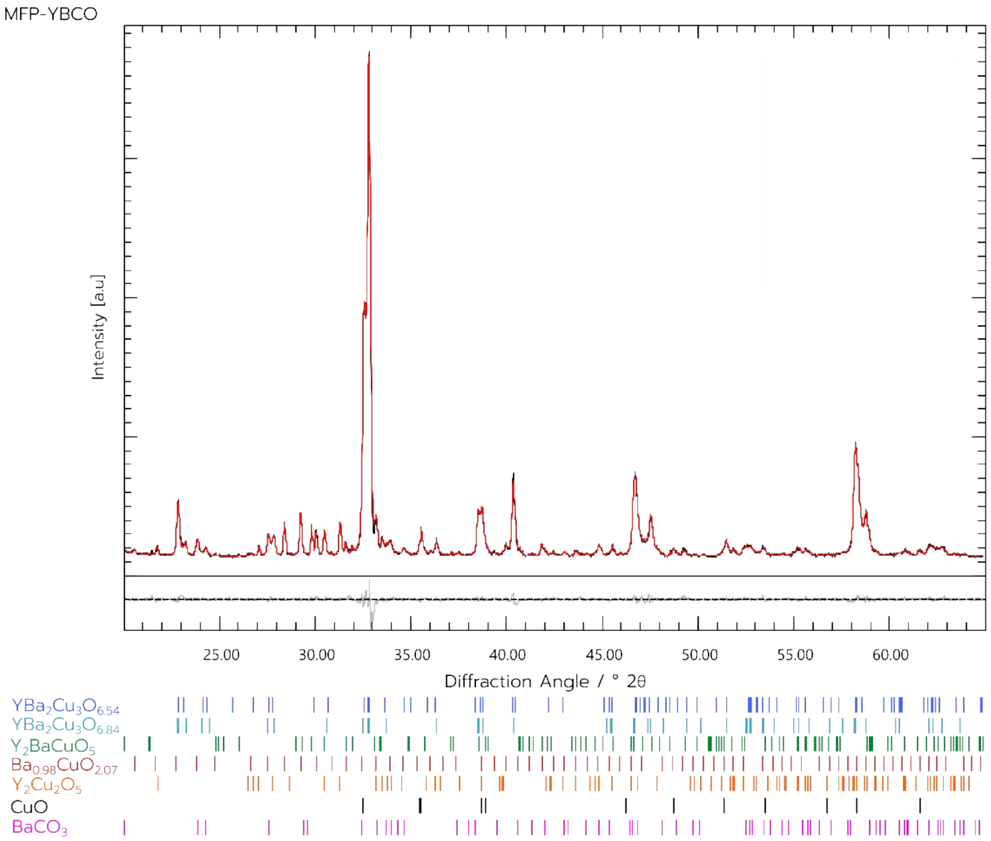


**Figure S3** – YBCO Pechini synthesis with the MF sponge (MFP-YBCO) refined PXRD pattern with $R_{wp}$ = 3.37 %. Observed and calculated plots are shown as black and red, respectively.

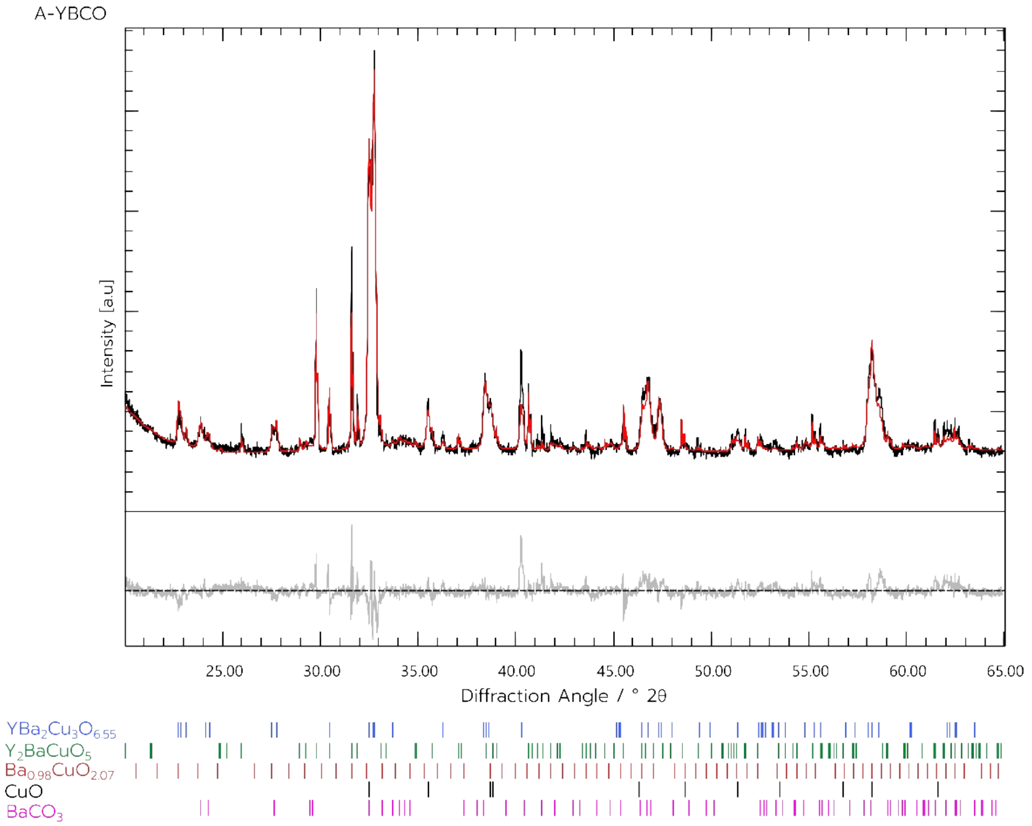


**Figure S4** - YBCO Alginate-only control synthesis $R_{wp}$ = 7.21 %. Observed and calculated plots are shown as black and red, respectively.

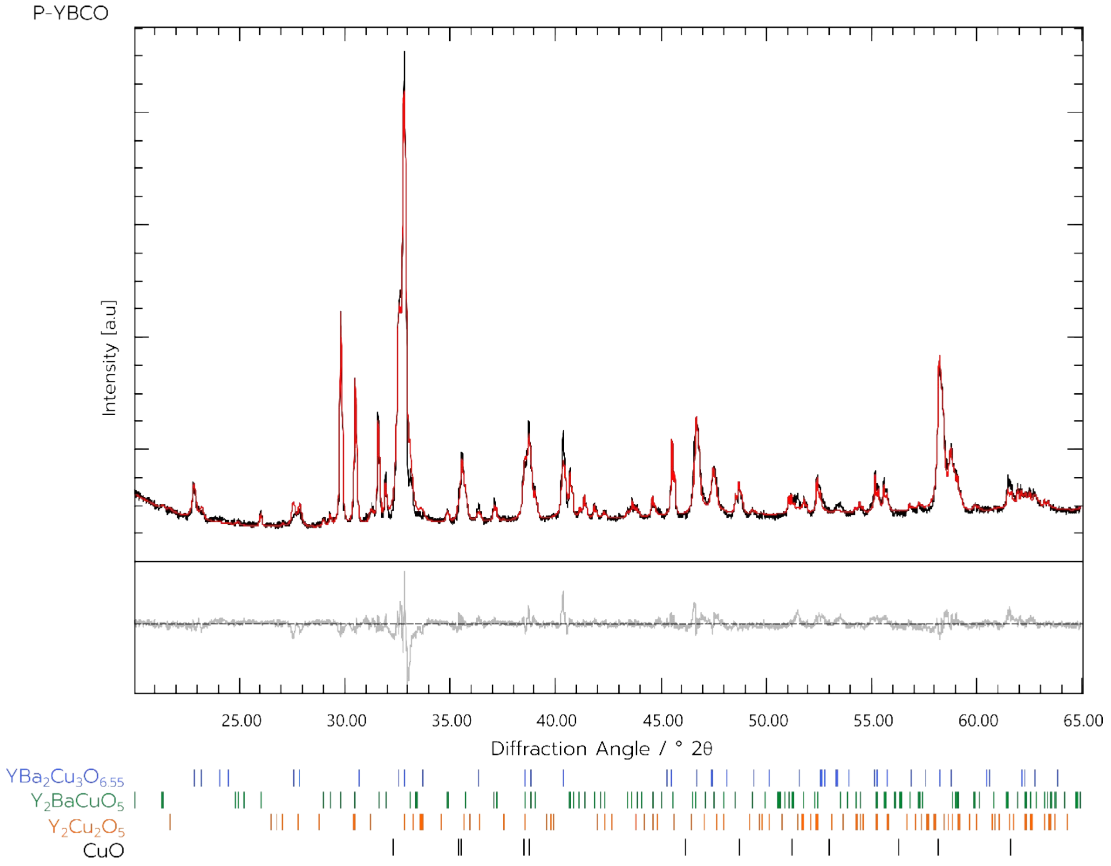


**Figure S5** – YBCO Pechini method control synthesis $R_{wp}$ = 6.45 %. Observed and calculated plots are shown as black and red, respectively.

| Phase | MF Only Quantity / % | Alginate method Quantity / % | Pechini method Quantity / % | Alginate only control Quantity / % | Pechini only control Quantity / % | ICSD reference code |
|---|---|---|---|---|---|---|
| $YBa_2Cu_3O_{6+\delta}$ | 36 | 65 | 69 | 59 | 50 | 79050 / 173898 / 83061 |
| $Y_2BaCuO_5$ | 17 | 13 | 4 | 18 | 22 | 10883 |
| $Ba_{0.98}CuO_{2.07}$ | 18 | 8 | 9 | 0 | 0 | 202998 |
| $Y_2Cu_2O_5$ | 4 | 1 | 8 | 0 | 4 | 72058 |
| CuO | 12 | 9 | 7 | 16 | 23 | 87126 |
| $BaCO_3$ | 4 | 4 | 3 | 6 | 0 | 15196 |
| $YBa_4Cu_3O_9$ | 9 | 0 | 0 | 0 | 0 | 65867 |

**Table S1** - Table of YBCO phases as determined by multi-phase Rietveld refinement

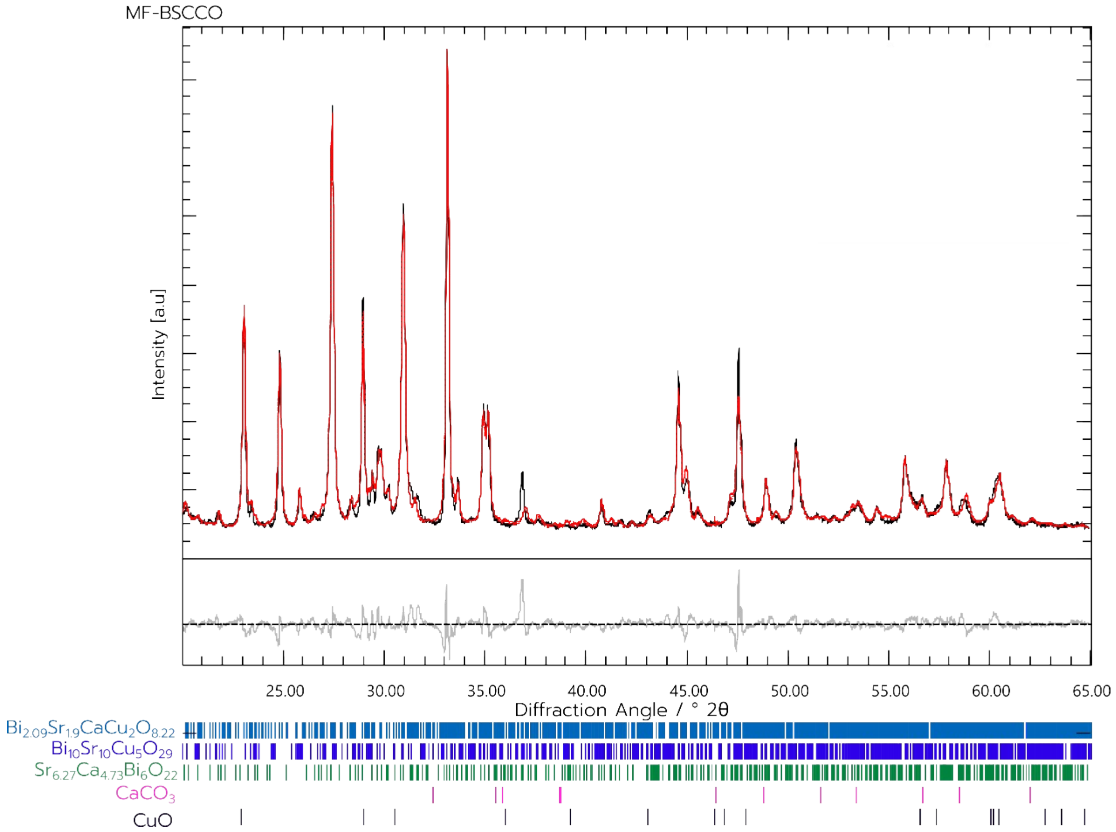


**Figure S6** - BSCCO MF sponge only (MF-BSCCO) refined PXRD pattern $R_{wp}$ = 8.93 %. Observed and calculated plots are shown as black and red, respectively.

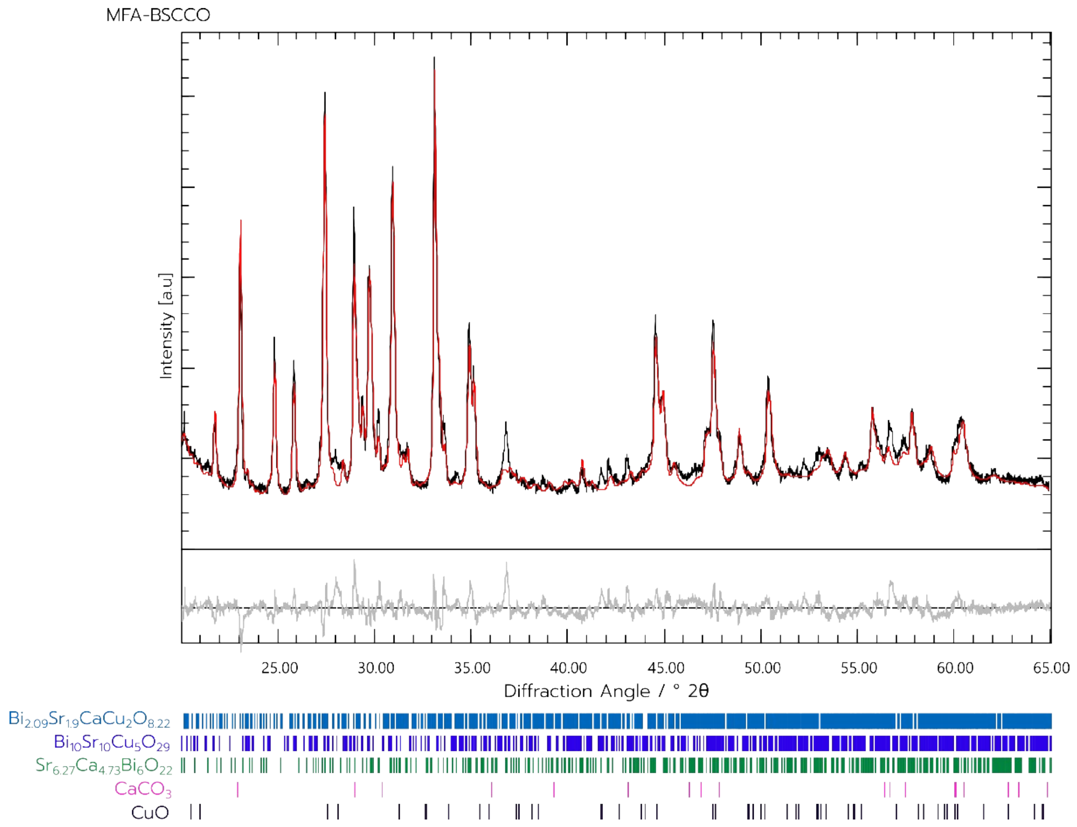


**Figure S7** - BSCCO alginate and MF sponge method (MFA-BSCCO) refined PXRD pattern, $R_{wp}$ = 7.72 %. Observed and calculated plots are shown as black and red, respectively.

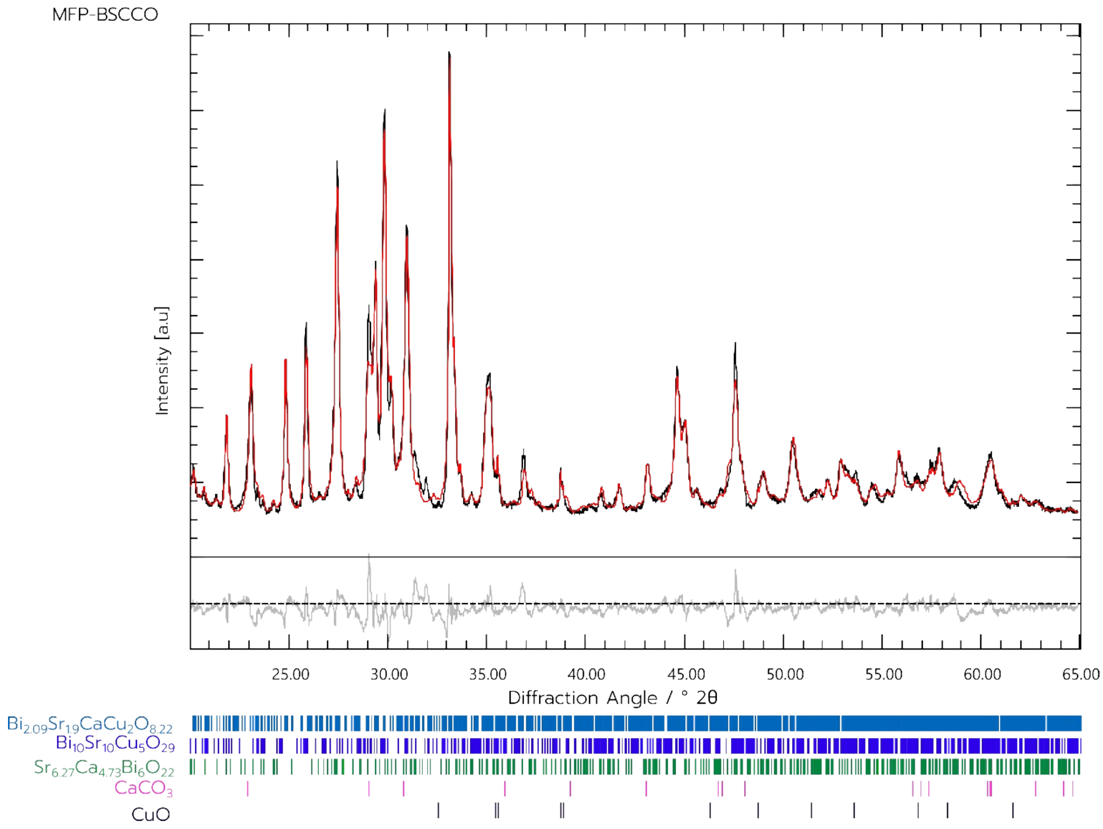


**Figure S8** - BSCCO Pechini synthesis with the MF sponge (MFP-BSCCO) refined PXRD pattern , $R_{wp}$ = 7.57 %. Observed and calculated plots are shown as black and red, respectively.

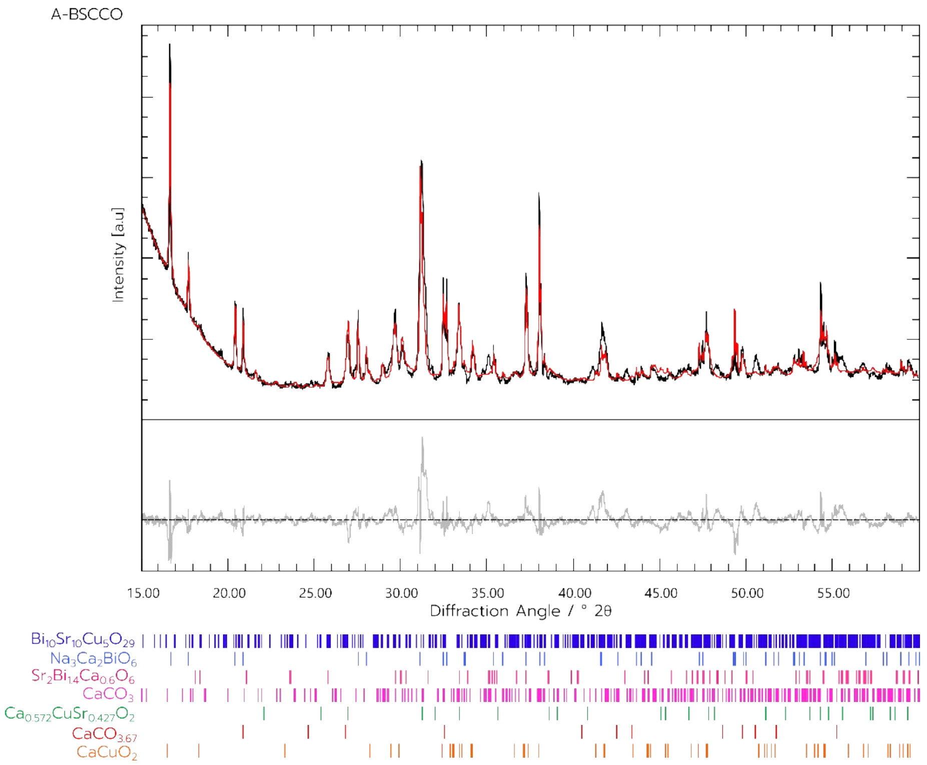


**Figure S9 –** BSCCO alginate only control synthesis $R_{wp}$ = 10.63 %. Observed and calculated plots are shown as black and red, respectively.

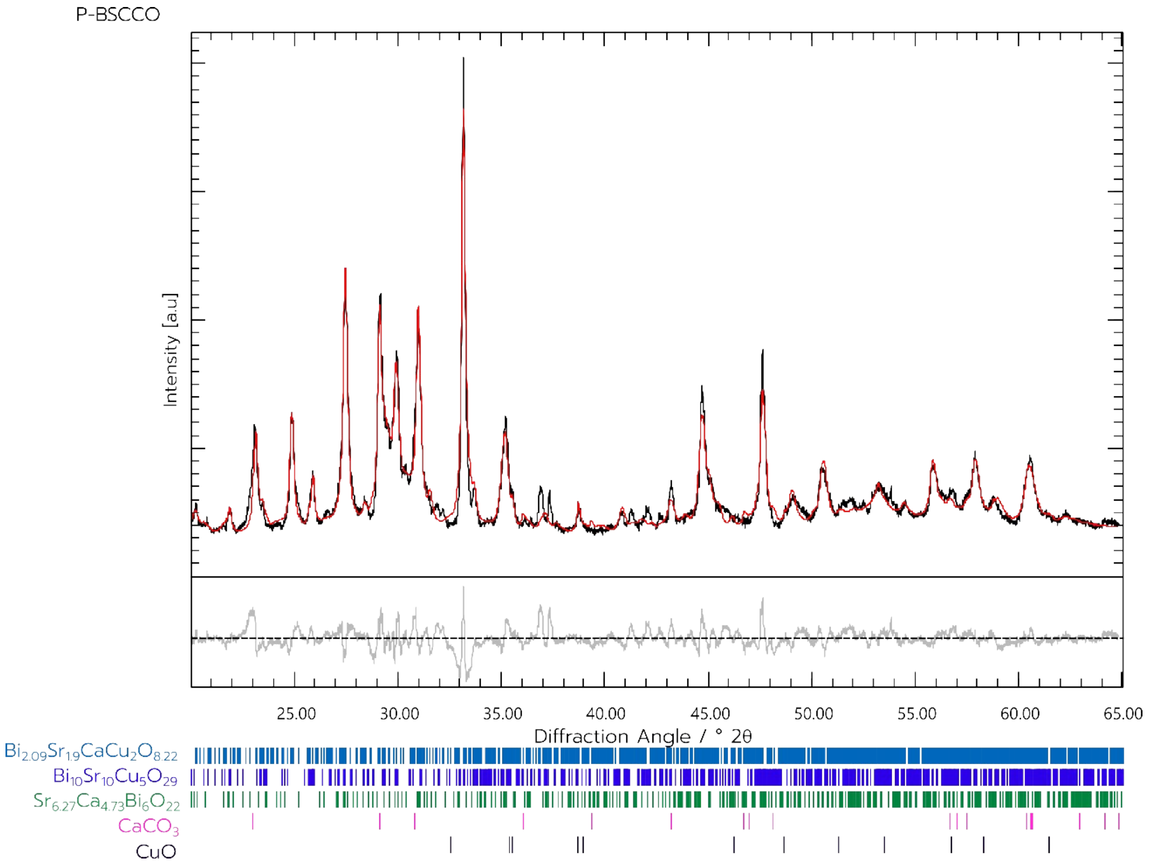


**Figure S10 –** BSCCO Pechini only control experiment $R_{wp}$ = 9.46 %. Observed and calculated plots are shown as black and red, respectively

| Phase | MF Only Quantity / % | Alginate method Quantity / % | Pechini method Quantity / % | Alginate only control Quantity / % | Pechini method only control Quantity / % | ICSD reference code |
|---|---|---|---|---|---|---|
| $Bi_{2.09}Sr_{0.9}CaCu_2O_{8.22}$ | 86 | 65 | 56 | - | 63 | 203210 |
| $Bi_{10}Sr_{10}Cu_5O_{29}$ | 6 | 17 | 17 | 5 | 6 | 65557 |
| $Sr_{6.27}Ca_{4.73}Bi_6O_{22}$ | 6 | 5 | 14 | - | 15 | 56774 |
| $CaCO_3$ | 2 | 14 | 11 | 44 | 12 | 191880 |
| CuO | 0 | 0 | 3 | - | 3 | 16025 |
| $Na_3Ca_2BiO_6$ | - | - | - | 23 | - | 240975 |
| $Ca_{0.573}CuO_2Sr_{0.427}$ | - | - | - | 6 | - | 77286 |
| $CaCuO_2$ | - | - | - | 17 | - | 84868 |
| $Ca(CO_3)_{0.67}$ | - | - | - | 6 | - | 130016 |

**Table S2 -** Table of BSCCO phases as determined by multi-phase Rietveld refinement

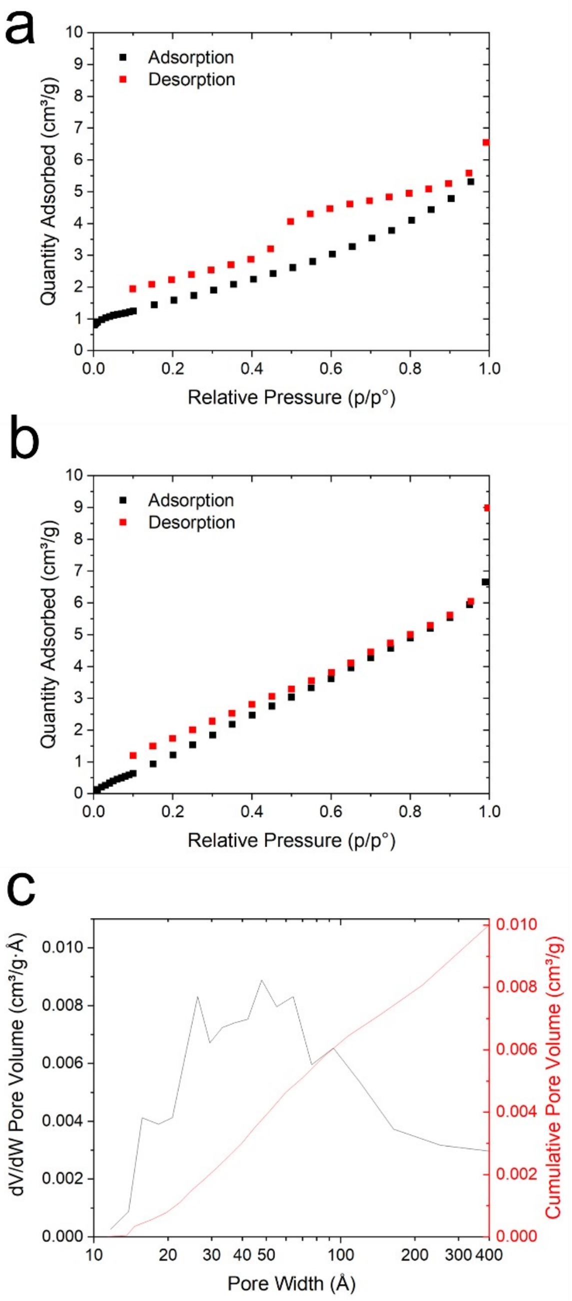


**Figure S11:** - $N_2$ gas sorption isotherms of a) MFP-YBCO sponges and b) MFP-BSCCO sponges. c) BJH pore size distribution for YBCO

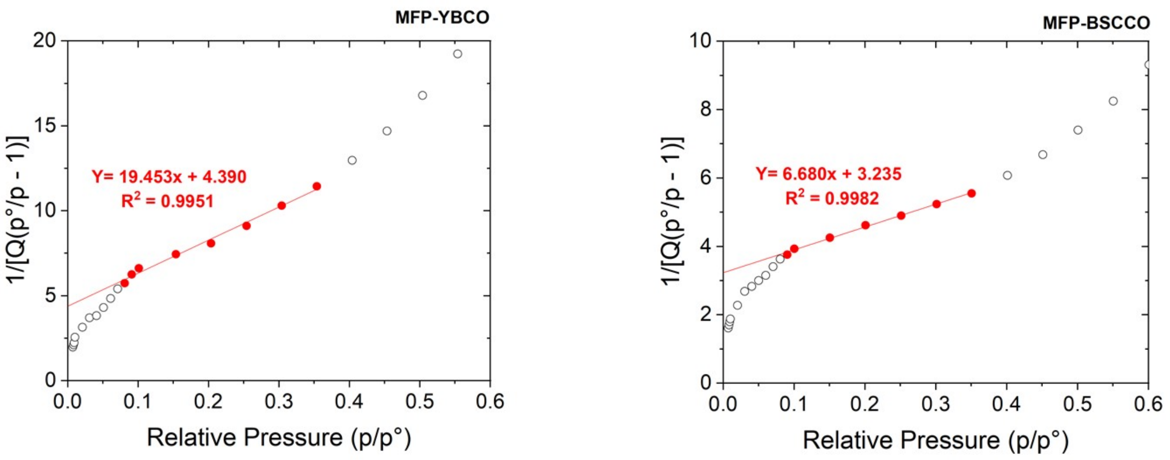


**Figure S12:** BET Plots for typical MFP-YBCO (A) and MFP-BSCCO (B) samples.
